# Plasma Xanthine Oxidoreductase Activity Is Associated with a High Risk of Cardiovascular Disease in a General Japanese Population

**DOI:** 10.3390/ijerph18041894

**Published:** 2021-02-16

**Authors:** Yuka Kotozaki, Mamoru Satoh, Kozo Tanno, Hideki Ohmomo, Ryo Otomo, Fumitaka Tanaka, Takahito Nasu, Satoru Taguchi, Hiroto Kikuchi, Takamasa Kobayashi, Atsushi Shimizu, Kiyomi Sakata, Jiro Hitomi, Kenji Sobue, Makoto Sasaki

**Affiliations:** 1 Iwate Tohoku Medical Megabank Organization, Iwate Medical University, Yahaba 028-3694, Japan; kotoyuka@iwate-med.ac.jp (Y.K.); ktanno@iwate-med.ac.jp (K.T.); hohmomo@iwate-med.ac.jp (H.O.); ryo.otomo@gmail.com (R.O.); ftanaka@iwate-med.ac.jp (F.T.); tnasu@iwate-med.ac.jp (T.N.); ashimizu@iwate-med.ac.jp (A.S.); ksakata@iwate-med.ac.jp (K.S.); jhitomi@iwate-med.ac.jp (J.H.); masasaki@iwate-med.ac.jp (M.S.); 2 Division of Biomedical Information Analysis, Institute for Biomedical Sciences, Iwate Medical University, Yahaba 028-3694, Japan; 3 Department of Hygiene and Preventive Medicine, Iwate Medical University, Yahaba 028-3694, Japan; 4 Division of Nephrology and Hypertension, Department of Internal Medicine, Iwate Medical University, Yahaba 028-3694, Japan; 5 Division of Cardiology, Department of Internal Medicine, Iwate Medical University, Yahaba 028-3694, Japan; staguchi@iwate-med.ac.jp (S.T.); apwgfka0174@gmail.com (H.K.); kesen3a08@yahoo.co.jp (T.K.); 6 Department of Anatomy, Iwate Medical University, Yahaba 028-3694, Japan; 7 Department of Neuroscience, Institute for Biomedical Sciences, Iwate Medical University, Yahaba 028-3694, Japan; ksobue@iwate-med.ac.jp; 8 Division of Ultrahigh field MRI, Institute for Biomedical Sciences, Iwate Medical University, Yahaba 028-3694, Japan

**Keywords:** atherosclerosis, Framingham Risk Score, the Iwate Tohoku Medical Megabank Organization, reactive oxygen species, uric acid

## Abstract

The purpose of this study was to investigate the association between xanthine oxidoreductase (XOR) activity and a high risk of cardiovascular disease (CVD) in a general Japanese population. The Iwate Tohoku Medical Megabank Organization pooled individual participant data from a general population-based cohort study in Iwate prefecture. The cardiovascular risk was calculated using the Framingham Risk Score (FRS). A total of 1605 of the 1631 participants (98.4%) had detectable XOR activity. Multiple regression analysis demonstrated that XOR activity was independently associated with body mass index (β = 0.26, *p* < 0.001), diabetes (β = 0.09, *p* < 0.001), dyslipidemia (β = 0.08, *p* = 0.001), and uric acid (β = 0.13, *p* < 0.001). Multivariate analysis showed that the highest quartile of XOR activity was associated with a high risk for CVD (FRS ≥ 15) after adjustment for baseline characteristics (OR 2.93, 95% CI 1.16–7.40). The area under the receiver operating characteristic curves of the FRS with XOR activity was 0.81 (*p* = 0.008). XOR activity is associated with a high risk for CVD, suggesting that high XOR activity may indicate cardiovascular risk in a general Japanese population.

## 1. Introduction

Xanthine oxidoreductase (XOR) is a ubiquitous uric acid (UA)-producing enzyme that catalyzes the oxidation of hypoxanthine to xanthine [1,2,3] and is the total activity of both xanthine dehydrogenase (XDH) and xanthine oxidase (XO) [4,5,6]. XDH reduces nicotinamide adenine dinucleotide, and XO consumes oxygen to produce superoxide (O_2_^−^) [1,5,6].

XOR activity and nicotinamide adenine dinucleotide phosphatase (NADPH) oxidase promote an increase in O_2_^−^ and is recognized as a significant source of reactive oxygen species (ROS) [5,7,8], contributing to the development of oxidative stress-related tissue injury [2,5,6,7,8]. An increase in XOR activity has been reported to be related to the development of various diseases, such as metabolic disorders and heart failure (HF) [9,10,11,12]. It has also been reported that XOR activity is involved in vascular inflammation and then the development of atherosclerosis [13]. In addition, NADPH oxidase plays an important role in atherosclerosis via ROS [7,8]. However, the association between XOR activity and the risk for cardiovascular disease (CVD), such as the Framingham Risk Score (FRS), has been uncertain in a general Japanese population.

The purpose of this study was to investigate the association between XOR activity and a high risk of CVD in a general Japanese population.

## 2. Materials and Methods

### 2.1. Study Population

The present study included 1737 participants (males/females = 577/1160) who participated in this study as part of a general population-based cohort study designed by the Iwate Tohoku Medical Megabank Organization (TMM). The participants were aged 20 years or older and completed self-administered questionnaires covering a wide range of topics, including sociodemographic factors, lifestyle habits, and a self-reported medical history. A history of hyperuricemia was defined based on the self-reported medical history. Blood samples were collected by experienced nurses. Participants were excluded from the study if they had a history of hyperuricemia, stroke, coronary artery disease (CAD), HF, or a malignant or primary wasting disorder.

In accordance with the Declaration of Helsinki (1991), written informed consent was obtained from each subject. This study was approved by the Ethics Committee of Iwate Medical University (HGH29-4).

### 2.2. Cohort Data Collection

All participants completed self-administered questionnaires and underwent standardized interviews conducted by trained research staff who collected information about medical history and medication. Blood samples and random spot urine samples were collected.

Hypertension was defined as systolic blood pressure (SBP) ≥140 mmHg, diastolic blood pressure (DBP) ≥90 mmHg, having been diagnosed with hypertension, and/or the use of antihypertensive medication [14]. Diabetes was defined as a glycated hemoglobin (HbA1c) value ≥6.5%, a non-fasting glucose concentration ≥200 mg/dL, having been diagnosed with diabetes, and/or undergoing treatment with antidiabetic drugs including insulin [15]. Dyslipidemia was defined as a low-density lipoprotein (LDL) cholesterol ≥140 mg/dL, having been diagnosed with dyslipidemia, and/or the use of antihyperlipidemic medication [16].

### 2.3. Measurements of XOR Activity

Plasma XOR activity was measured using frozen samples that were maintained at −80 °C until the time of assay and was measured using the recently established assay using stable isotope-labelled [13C2,15N2] xanthine with liquid chromatography mass spectrometry (Nano Space SI-2 LC system, Shiseido, Tokyo, Japan) and a TSQ-Quantum triple quadrupole mass spectrometer (Thermo Fisher Scientific GmbH, Bremen, Germany) [17,18]. The calibration curve of [13C2,15N2] UA showed linearity over the range of 4–4000 nM (r^2^ > 0.995) with a lower limit of quantitation of 4 nM. The lower detection limit of XOR activity was 6.67 pmol/h/mL plasma, and intra- and inter-assay coefficients of variation of human plasma XOR activity were 6.5% and 9.1%, respectively [17].

### 2.4. Framingham Risk Score

The FRS includes gender, age, total cholesterol value, HDL-cholesterol value, SBP, presence or absence of diabetes, and the presence or absence of smoking [19]. The FRS can predict the development of ischemic heart disease within 10 years [19]. In this study, we classified a high risk of CVD when FRS was ≥15 based on a previous study [20].

### 2.5. Statistical Analysis

Numeric variables are presented as mean ± standard deviation (SD) for normal distribution and median (interquartile range) for skewed variables. Categorical data are presented as frequencies and percentages. Student’s *t*-test for continuous variables that exhibited a normal distribution, Mann–Whitney U test for continuous variables with skewed variables, and a chi-square test for categorical variables were used to evaluate differences in characteristics. Non-normally distributed parameters were logarithmically transformed for the analysis. The correlation between two variables was evaluated using Pearson’s correlation coefficient. We performed multiple regressions to identify independent determinants of XOR activity using age, gender, and the variables with significance after consideration of multicollinearity. Additionally, the distribution of XOR activity was confirmed and classified into quartiles. The basic attributes were compared among the four groups by using analysis of variance (ANOVA) for normal distribution and the Kruskal–Wallis test for skewed variables. Additionally, we performed a logistic regression to identify the association between XOR activity and a high risk for CVD, and we obtained receiver operating characteristic (ROC) curves to examine the strength of the correlation between a high risk of CVD and XOR activity. All data were analyzed using IBM SPSS Statistics version 25 for Windows (IBM Corp., Armonk, NY, USA). Differences of *p* < 0.05 were considered statistically significant.

## 3. Results

### 3.1. Baseline Characteristics of the Study Population

The baseline characteristics of the study participants are shown in Table 1. XOR activity was measured in a total of 1737 participants. After applying exclusion criteria, we excluded 106 participants from the analysis. A total of 1631 volunteers who participated in the TMM Community-Based Cohort Study were included in the present analyses. Significant differences in age, body mass index (BMI), current smoking, current drinking, alcohol consumption, SBP, and DBP were found between males and females. Hypertension and diabetes were more prevalent in males than in females. Significant differences in UA, aspartate aminotransferase (AST), alanine aminotransferase (ALT), LDL-cholesterol, HDL-cholesterol, total cholesterol, and HbA1c in laboratory data were also observed between males and females.

### 3.2. Concentration and Distribution of XOR Activity

**Figure 1:**
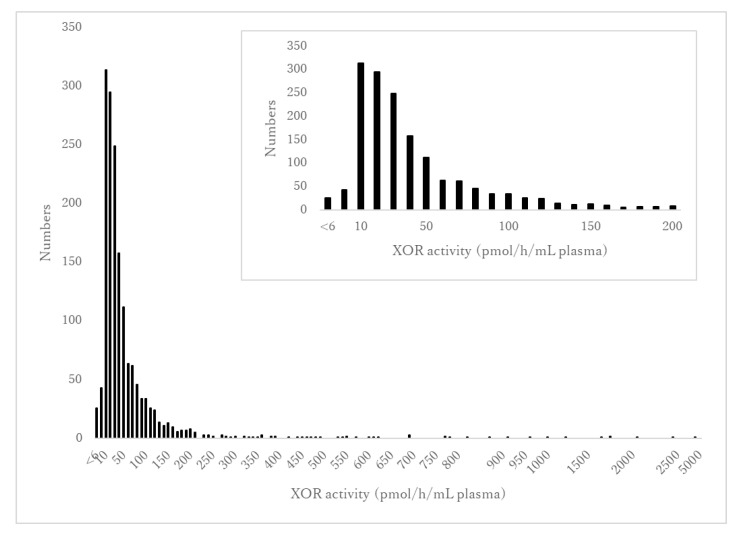
The distribution of XOR activity. The vertical axis indicates the number of participants. The horizontal axis shows XOR activity levels ranging from <6 to 4540 pmol/h/mL plasma in increments of 50. The inset figure shows an enlarged version of the distribution of XOR activity in the range from <6 to 200 pmol/h/mL plasma.

The distribution of XOR activity is shown in Figure 1. The range of detectable XOR activity was 5–4540 pmol/h/mL plasma with a median value of 34.8 pmol/h/mL plasma. A total of 26 of the 1631 participants (1.6%) had undetectable XOR activity (<6.7 pmol/h/mL plasma) while 1605 participants (98.4%) had detectable XOR activity (≥6.7 pmol/h/mL plasma). There was a significant difference in XOR activity between males and females (Table 1). There was no significant difference in XOR activity between without and with alcohol consumption (34.7 (20.7–63.0) vs. 49.3 (33.5–59.0) pmol/h/mL plasma, *p* = 0.053).

### 3.3. Association between XOR Activity and Baseline Characteristics

The correlation between XOR activity and other variables is shown in Table 2. XOR activity was weakly positively correlated with BMI (r = 0.33, *p* < 0.001), UA (r = 0.22, *p* < 0.001), AST (r = 0.58, *p* < 0.001), ALT (r = 0.68, *p* < 0.001), and HbA1c (r = 0.25, *p* < 0.001).

Multiple regression analyses are shown in Table 3. When the multiple regression analysis was performed using the liver enzyme including AST and ALT, multicollinearity (VIF: AST = 3.579, ALT = 3.759) occurred in this model. The present study therefore removed both variables in the multiple regression analysis. XOR activity was independently associated with BMI (β = 0.26, *p* < 0.001), diabetes (β = 0.09, *p* < 0.001), dyslipidemia (β = 0.08, *p* = 0.001), and UA (β = 0.13, *p* < 0.001) (R^2^ = 0.44, *p* < 0.001).

### 3.4. Comparisons of FRS among Participants with Hypertension, Diabetes, or Dyslipidemia and Those without Them

The FRS scores were higher for participants with hypertension, diabetes, or dyslipidemia compared with those without them (Table 4).

### 3.5. Comparisons between XOR Activity and Baseline Characteristics

The baseline characteristics were classified into quartiles of XOR activity (Table 5). The prevalence of hypertension, diabetes, and dyslipidemia and baseline characteristics including age, BMI, FRS, SDP, DBP, UA, AST, ALT, and HbA1c in Q4 were higher than those in Q1–3 (*p* < 0.001).

### 3.6. XOR Activity for the Identification of High Risk for CVD

**Figure 2:**
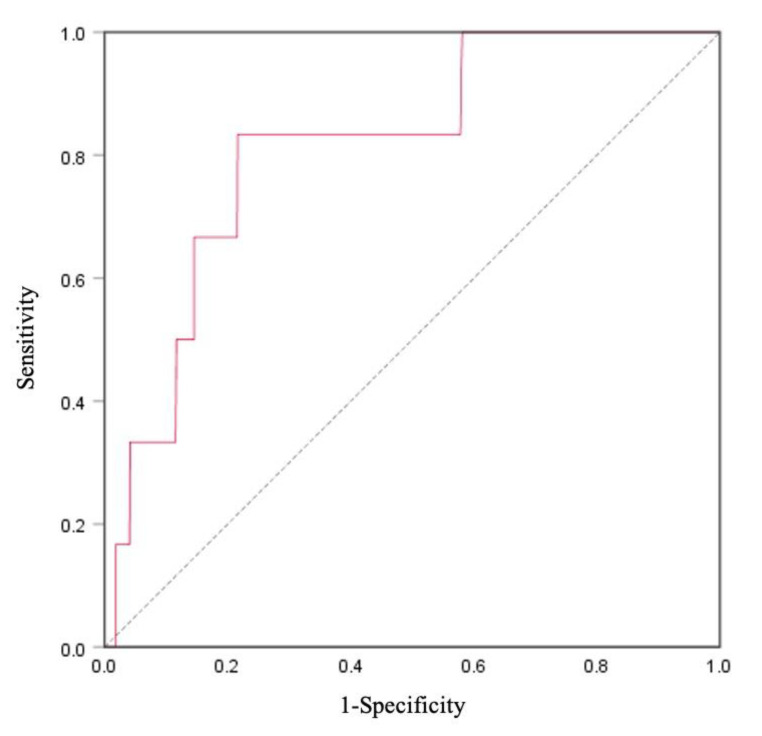
Receiver operating characteristic (ROC) curve of Framingham Risk Score (FRS) and XOR activity for cardiovascular disease (CVD) prediction in total participants (*n* = 1631). The area under the curve (AUC) of CVD was 0.81 (95% CI = 0.66–0.97), *p* = 0.008.

The logistic regression analyses are shown in Table 6. The odds ratios and 95% confidence intervals (CIs) of the high risk for CVD (FRS ≥ 15) were 2.93 (95% CI = 1.16−7.40, *p* = 0.023). ROC curve analysis for high risk for CVD in XOR activity is shown in Figure 2. The area under the curve (AUC) was found to be 0.81 (95% CI = 0.66−0.97, *p* = 0.008).

## 4. Discussion

The present study investigated the association between XOR activity and a high risk for CVD in a general Japanese population. XOR activity was significantly and independently associated with BMI, diabetes, dyslipidemia, and UA. In addition, XOR activity was strongly related to a high risk of CVD. To the best of our knowledge, this is the first study to reveal the association between XOR activity and the risk of CVD in the general Japanese population.

Our study showed a significant difference in XOR activity between males and females. Similar to our study, a previous study on the general Japanese population found higher XOR activity in males than in females [12]. Baseline characteristics showed significant differences in laboratory data and the prevalence of smoking and medical history between males and females. It has therefore been suggested that the gender difference of XOR activity may be related to the prevalence of smoking and medical history, including hypertension, diabetes, and dyslipidemia.

Multiple regression analyses showed that XOR activity was independently associated with the presence of diabetes and dyslipidemia. In addition, XOR activity was positively correlated with UA and HbA1c levels. It has been reported that XOR activity is positively correlated with HbA1c in diabetic patients [21]. Furuhashi et al., in their study on a general Japanese population, reported that XOR activity was associated with dyslipidemia [12]. An elevated XOR activity is responsible for the formation of UA from hypoxanthine and xanthine, leading to O_2_^−^ and ROS production [4,22]. It is well known that ROS production in the vessel wall is involved in the progression of arteriosclerosis [23]. XOR activity generates ROS and causes endothelial dysfunction [2,3,6,24,25,26]. In addition, XOR activity has been known to reflect the degree of progression of arteriosclerosis [2,3]. A study on outpatients with CVD also reported that higher XOR activity was independently associated with diabetes [27]. These observations suggest that the presence of diabetes and dyslipidemia may induce ROS production via elevated XOR activity and then be related to the progression of atherosclerosis.

The present study showed that XOR activity was positively correlated with BMI. It has been demonstrated before that XOR activity is correlated with obesity [3]. An experimental study has previously shown that adipose tissues in obese mice have higher XOR activities than control mice [8]. In addition, adipose tissue is an important determinant of the chronic inflammatory state, as reflected by levels of proinflammatory cytokines, suggesting a link between the latter and obesity and CVD [28]. It has also been reported that XOR activity is positively correlated with BMI and subclinical inflammation in young humans [29]. These observations suggest that adiposity is closely associated with XOR activity and is involved in the progression of CVD through vascular inflammation.

The FRS is a common tool for predicting the likelihood of developing CVD in the long term, and a high FRS indicates a high risk of future CVD events [19]. Our results showed that elevated XOR activity was significantly related to high FRS, indicating a high risk for CVD. In addition, the AUC was 0.81 (95% CI = 0.66–0.97), suggesting a high predictive and diagnostic ability of XOR as the biomarker for the risk of CVD. NADPH oxidase and XOR activities contribute to the generation of ROS and oxidative stress-related tissue damage [2,5,6,7,8], leading to the development of CVD [6]. From these observations, it has been speculated that XOR activity may serve as a new biomarker for predicting future CVD.

There are a few limitations to this study while interpreting our results. First, only a baseline measurement of XOR activity and other covariates was performed because this was a cross-sectional study. Second, this study evaluated whether each participant had CVD using a self-reported questionnaire rather than clinical examination. A future prospective study is needed to determine whether XOR activity is an independent predictor of long-term cardiovascular outcomes. Third, although NADPH oxidase plays an important role in oxidative stress and progression of atherosclerosis [7,8], the present study did not measure NADPH oxidase levels owing to a lack of funds and samples.

## 5. Conclusions

The present study showed that XOR activity is associated with a high risk for CVD, suggesting that elevated XOR activity may indicate cardiovascular risk in a general Japanese population.

## Figures and Tables

**Table 1:** The characteristics of participants.

	Total	Males	Females	*p* Value
No. of participants	1631	510	1121	
Age (years old)	66.1 ± 9.6	69.1 ± 7.4	64.7 ± 10.1	<0.001
BMI (kg/m^2^)	23.7 ± 9.4	23.9 ± 2.9	23.1 ± 3.6	<0.001
Current smoking (%)	8.9	20.1	4.0	<0.001
Current drinking (%)	40.7	71.1	27.3	<0.001
Alcohol consumption (%)	1.3	4.3	0	<0.001
Medical history				
Hypertension (%)	46.2	53.1	43.1	<0.001
Diabetes (%)	8.9	13.5	6.8	<0.001
Dyslipidemia (%)	41.8	39.4	42.8	0.196
Framingham Risk Score ≥ 15 (%)	3.5	9.2	0.9	<0.001
SBP (mmHg)	133.7 ± 18.8	136.0 ± 17.6	132.6 ± 19.2	0.001
DBP (mmHg)	74.3 ± 10.8	76.3 ± 10.9	73.4 ± 10.6	<0.001
XOR (pmol/h/mL plasma)	34.8 (20.8–62.7)	43.7 (25.3–77.3)	31.6 (19.5–58.4)	<0.001
UA (mg/mL)	5.0 ± 4.9	5.9 ± 1.2	4.6 ± 1.0	<0.001
AST (IU/L)	23.0 (20.0–27.0)	24.0 (21.0–30.0)	24.2 (20.0–26.0)	<0.001
ALT (IU/L)	18.0 (14.0–23.0)	20.0 (16.0–26.0)	19.6 (14.0–22.0)	<0.001
LDL-cholesterol (mg/dL)	114.6 ± 28.1	107.9 ± 26.2	117.6 ± 28.5	<0.001
HDL-cholesterol (mg/dL)	61.7 ± 14.5	56.4 ± 14.1	64.1 ± 14.1	<0.001
Total-choresterol (mg/dL)	208.8 ± 34.5	196.5 ± 31.3	214.4 ± 34.5	<0.001
HbA1c (%)	5.6 ± 0.6	5.7 ± 0.7	5.6 ± 0.5	0.005

Statistical significant (males vs. females, *p* < 0.05). BMI, Body Mass Index; SBP, systolic blood pressure; DBP, diastolic blood pressure; XOR, Xanthine oxidoreductase; UA, Urid acid; AST, aspartate aminotransferase; ALT, alanine aminotransferase; LDL, low density lipoprotein; HDL, high density lipoprotein; HbA1c, glycated hemoglobin A1c. Student’s *t*-test was used for comparisons of continuous variables that exhibited a normal distribution. Mann-Whitney U test was used for comparisons of continuous variables with a skewed distribution. Chi-square test was used for comparisons of categorical variables.

**Table 2:** The correlation between Log10XOR activity.

	r	*p* Value
Age	0.10	<0.001
Gender	−0.11	<0.001
BMI	0.33	<0.001
SBP	0.15	<0.001
DBP	0.14	<0.001
Log10UA	0.22	<0.001
Log10AST	0.58	<0.001
Log10ALT	0.68	<0.001
LDL-cholesterol	0.03	0.304
HDL-cholesterol	−0.19	<0.001
Total-cholesterol	−0.03	0.315
HbA1c	0.25	<0.001

Statistical significant (*p* < 0.05). BMI, Body Mass Index; SBP, systolic blood pressure; DBP, diastolic blood pressure; UA, Urid acid; AST, aspartate aminotransferase; ALT, alanine aminotransferase; LDL, low density lipoprotein; HDL, high density lipoprotein; HbA1c, glycated hemoglobin A1c.

**Table 3:** Multivariate regression analysis for Log10XOR activity.

	β	*p* Value
Age	0.04	0.081
Gender	−0.01	0.772
BMI	0.26	<0.001
Hypertension	0.03	0.188
Diabetes	0.09	<0.001
Dyslipidemia	0.08	0.001
Alcohol consumption	0.01	0.811
Log10UA	0.13	<0.001

Statistical significant (*p* < 0.05). R^2^ = 0.44, *p* < 0.001 β: standardized correlation coefficient, R^2^: multiple coefficient of determination. BMI, Body Mass Index; UA, Urid acid.

**Table 4:** Comparisons of FRS among participants with hypertension, diabetes, or dyslipidemia and those without them.

	No of Subjects	FRS	*p* Value
Hypertension			
+	754	7.0 ± 4.2	<0.001
−	877	4.1 ± 2.9	
Diabetes			
+	145	11.9 ± 6.6	<0.001
−	1486	4.8 ± 2.8	
Dyslipidemia			
+	681	6.4 ± 4.4	<0.001
−	950	4.7 ± 3.3	

Statistical significant (*p* < 0.05). FRS, Framingham Risk Score. Mann-Whitney U test.

**Table 5:** The baseline characteristics were classified into quartiles of XOR activity.

XOR Activity Quartile (pmol/h/mL Plasma)	Q1 (–20.80)	Q2 (20.81–34.80)	Q3 (34.81–62.70)	Q4 (62.71–)	*p* Value
No. of participants	411	405	408	407	
Age (years old)	64.2 ± 11.5	65.9 ± 9.7	67.1 ± 8.6	67.3 ± 7.8	<0.001
Gender (female, %)	76.6	73.8	62.3	62.2	<0.001
BMI (kg/m^2^)	22.1 ± 3.0	22.8 ± 2.9	23.6 ± 3.3	24.9 ± 3.8	<0.001
Current smoking (%)	8.3	10.1	8.6	8.7	0.906
Current drinkng (%)	34.1	40.2	44.1	43.5	0.134
Alcohol consumption (%)	0.7	0.7	2.9	1.0	0.015
Current drinking (%)	34.1	40.2	44.1	43.5	0.134
Medical history					
Hypertension (%)	37.0	44.7	49.8	53.6	<0.001
Diabetes (%)	4.9	6.2	8.6	16.0	<0.001
Dyslipidemia (%)	32.6	40.7	41.9	51.8	<0.001
Framingham Risk Score ≥ 15 (%)	1.5	2.5	3.4	6.6	<0.001
SBP (mmHg)	130.0 ± 19.7	132.7 ± 18.9	135.2 ± 17.3	136.8 ± 18.5	<0.001
DBP (mmHg)	72.4 ± 11.0	73.9 ± 10.8	74.9 ± 10.2	75.8 ± 11.0	<0.001
UA (mg/mL)	4.7 ± 1.2	4.8 ± 1.1	5.3 ± 1.2	5.3 ± 1.2	<0.001
AST (IU/L)	21.0 (18.0–23.0)	22.0 (19.0–25.0)	24.0 (21.0–27.0)	28.0 (24.0–34.0)	<0.001
ALT (IU/L)	14.0 (12.0–17.0)	16.0 (14.0–19.0)	19.0 (16.0–23.0)	26.0 (20.0–35.0)	<0.001
LDL-cholesterol (mg/dL)	113.8 ± 26.3	114.8 ± 27.0	114.8 ± 29.4	114.9 ± 29.7	0.932
HDL-cholesterol (mg/dL)	64.2 ± 14.9	64.0 ± 14.8	61.7 ± 13.9	56.9 ± 13.3	<0.001
Total-cholesterol (mg/dL)	209.0 ± 34.0	210.8 ± 32.5	210.0 ± 35.3	205.7 ± 36.1	0.169
HbA1c (%)	5.5 ± 0.4	5.6 ± 0.5	5.6 ± 0.5	5.8 ± 0.7	<0.001

Statistical significant (*p* < 0.05). BMI, Body Mass Index; SBP, systolic blood pressure; DBP, diastolic blood pressure; XOR, Xan thine oxidoreductase; UA, Urid acid; AST, aspartate aminotransferase; ALT, alanine aminotransferase; LDL, low density lipoprotein; HDL, high density lipoprotein; HbA1c, glycated hemoglobin A1c. Analysis of variance was used for comparisons of continuous variables that exhibited a normal distribution. Kruskal-Wallis test was used for comparisons of continuous variables with a skewed distribution. Chi-square test was used for comparisons of categorical variables.

**Table 6:** Multivariate adjusted ORs for the high risk for CVD (FRS ≥ 15) by XOR quartiles.

XOR Quartiles	OR	95% CI	*p* Value
Q1	Reference		
Q2	1.56	0.56–4.35	0.396
Q3	1.68	0.63–4.50	0.298
Q4	2.93	1.16–7.40	0.023

Statistical significant (*p* < 0.05). OR, Odd ratios; 95% CI, 95% confidence intervals. Covariates: BMI, Log10UA.

## Data Availability

Data sharing is not applicable to this article due to restrictions e.g., privacy or ethical.

## References

[1] Nishino T. (1994). The conversion of xanthine dehydrogenase to xanthine oxidase and the role of the enzyme in reperfusion injury. J. Biochem..

[2] Battelli M.G., Bolognesi A., Polito L. (2014). Pathophysiology of circulating xanthine oxidoreductase: New emerging roles for a multi-tasking enzyme. Biochim. Biophys. Acta.

[3] Battelli M.G., Polito L., Bolognesi A. (2014). Xanthine oxidoreductase in atherosclerosis pathogenesis: Not only oxidative stress. Atherosclerosis.

[4] Amaya Y., Yamazaki K., Sato M., Noda K., Nishino T., Nishino T. (1990). Proteolytic conversion of xanthine dehydrogenase from the NAD-dependent type to the O2-dependent type. Amino acid sequence of rat liver xanthine dehydrogenase and identification of the cleavage sites of the enzyme protein during irreversible conversion by trypsin. J. Biol. Chem..

[5] Meneshian A., Bulkley G.B. (2002). The physiology of endothelial xanthine oxidase: From urate catabolism to reperfusion injury to inflammatory signal transduction. Microcirculation.

[6] Berry C.E., Hare J.M. (2004). Xanthine oxidoreductase and cardiovascular disease: Molecular mechanisms and pathophysiological implications. J. Physiol..

[7] Paravicini T.M., Touyz R.M. (2008). NADPH oxidases, reactive oxygen species, and hypertension: Clinical implications and therapeutic possibilities. Diabetes Care.

[8] Drummond G.R., Selemidis S., Griendling K.K., Sobey C.G. (2011). Combating oxidative stress in vascular disease: NADPH oxidases as therapeutic targets. Nat. Rev. Drug Discov..

[9] Fujimura Y., Yamauchi Y., Murase T., Nakamura T., Fujita S.I., Fujisaka T., Ito T., Sohmiya K., Hoshiga M., Ishizaka N. (2017). Relationship between plasma xanthine oxidoreductase activity and left ventricular ejection fraction and hypertrophy among cardiac patients. PLoS ONE.

[10] Nakatani A., Nakatani S., Ishimura E., Murase T., Nakamura T., Sakura M., Tateishi Y., Tsuda A., Kurajoh M., Moru K. (2017). Xanthine oxidoreductase activity is associated with serum uric acid and glycemic control in hemodialysis patients. Sci. Rep..

[11] Otaki Y., Watanabe T., Kinoshita D., Yokoyama M., Takahashi T., Toshima T., Sugai T., Murase T., Nakamura T., Nishiyama S. (2017). Association of plasma xanthine oxidoreductase activity with severity and clinical outcome in patients with chronic heart failure. Int. J. Cardiol..

[12] Furuhashi M., Matsumoto M., Tanaka M., Moniwa N., Murase T., Nakamura T., Ohnishi H., Saitoh S., Shimamoto K., Miura T. (2018). Plasma xanthine oxidoreductase activity as a novel biomarker of metabolic disorders in a general population. Circ. J..

[13] Kushiyama A., Okubo H., Sakoda H., Kikuchi T., Fujishiro M., Sato H., Kushiyama S., Iwashita M., Nishimura F., Fukushima T. (2012). Xanthine oxidoreductase is involved in macrophage foam cell formation and atherosclerosis development. Arterioscler. Thromb. Vasc. Biol..

[14] Shimamoto K., Ando K., Fujita T., Hasebe N., Higaki J., Horiuchi M., Imai Y., Imaizumi T., Ishimitsu T., Ito M. (2014). Japanese Society of Hypertension Committee for Guidelines for the Management of Hypertension. The Japanese society of hypertension guidelines for the management of hypertension (JSH 2014). Hypertens. Res..

[15] Haneda M., Noda M., Origasa H., Noto H., Yabe D., Fujita Y., Goto A., Kondo T., Araki E. (2018). Japanese clinical practice guideline for diabetes 2016. Diabetol. Int..

[16] Kinoshita M., Yokote K., Arai H., Iida M., Ishigaki Y., Ishibashi S., Umemoto S., Egusa G., Ohmura H., Okamura T. (2018). Japan atherosclerosis society (JAS) guidelines for prevention of atherosclerotic cardiovascular diseases 2017. J. Atheroscler. Thromb..

[17] Murase T., Nampei M., Oka M., Miyachi A., Nakamura T. (2016). A highly sensitive assay of human plasma xanthine oxidoreductase activity using stable isotope-labeled xanthine and LC/TQMS. J. Chromatogr. B Anal. Technol. Biomed. Life Sci..

[18] Murase T., Oka M., Nampei M., Miyachi A., Nakamura T. (2016). A highly sensitive assay for xanthine oxidoreductase activity using a combination of [(13) C2, (15) N2]xanthine and liquid chromatography/triple quadrupole mass spectrometry. J. Label. Comp. Radiopharm..

[19] Wilson P.W., D’Agostino R.B., Levy D., Belanger A.M., Silbershatz H., Kannel W.B. (1998). Prediction of coronary heart disease using risk factor categories. Circulation.

[20] Wright J.T., Williamson J.D., Whelton P.K., Snyder J.K., Sink K.M., Rocco M.V., Reboussin D.M., Rahman M., Oparil S., SPRINT Research Group (2015). A randomized trial of intensive versus standard blood-pressure control. N. Engl. J. Med..

[21] Kuppusamy U.R., Indran M., Rokiah P. (2005). Glycaemic control in relation to xanthine oxidase and antioxidant indices in Malaysian Type 2 diabetes patients. Diabet. Med..

[22] Suvorava T., Kojda G. (2009). Reactive oxygen species as cardiovascular mediators: Lessons from endothelial-specific protein overexpression mouse models. Biochim. Biophys. Acta.

[23] Förstermann U., Xia N., Li H. (2017). Roles of vascular oxidative stress and nitric oxide in the pathogenesis of atherosclerosis. Circ. Res..

[24] Kelley E.E. (2015). A new paradigm for XOR-catalyzed reactive species generation in the endothelium. Pharmacol. Rep..

[25] Tsushima Y., Nishizawa H., Tochino Y., Nakatsuji H., Sekimoto R., Nagao H., Shirakura T., Kato K., Imaizumi K., Takahashi H. (2013). Uric acid secretion from mouse adipose tissue uric acid secretion from adipose tissue and its increase in obesity. J. Biol. Chem..

[26] Battelli M.G., Polito L., Bortolotti M., Bolognesi A. (2016). Xanthine oxidoreductase-derived reactive species: Physiological and pathological effects. Oxid. Med. Cell. Longev..

[27] Matsushita M., Murase T., Nakamura T., Takayasu T., Asano M., Okajima F., Kobayashi N., Hata N., Asai K., Shimizu W. (2020). Plasma xanthine oxidoreductase (XOR) activity in cardiovascular disease outpatients. Circ. Rep..

[28] Yudkin J.S., Stehouwer C.D., Emeis J.J., Coppack S.W. (1999). C-reactive protein in healthy subjects: Associations with obesity; insulin resistance; and endothelial dysfunction: A potential role for cytokines originating from adipose tissue?. Arterioscler. Thromb. Vasc. Biol..

[29] Washio K.W., Kusunoki Y., Murase T., Nakamura T., Osugi K., Ohigashi M., Sukenaga T., Ochi F., Matsuo T., Katsuno T. (2017). Xanthine oxidoreductase activity is correlated with insulin resistance and subclinical inflammation in young humans. Metabolism.

